# Ileocolic intussusception as the initial presentation of Burkitt lymphoma in a 27-year-old male: a case report

**DOI:** 10.1093/jscr/rjaf460

**Published:** 2025-07-01

**Authors:** Jacob E Chinthagada, Patrick T Gorman, Robert R Fogle, Nadeem Kutaish, David Diep

**Affiliations:** University of Toledo College of Medicine and Life Sciences, 3000 Arlington Avenue, Toledo, OH 43614, United States; University of Toledo College of Medicine and Life Sciences, 3000 Arlington Avenue, Toledo, OH 43614, United States; University of Toledo College of Medicine and Life Sciences, 3000 Arlington Avenue, Toledo, OH 43614, United States; Promedica Pathology, Consultants in Laboratory Medicine, 2130 West Central Avenue, Toledo, OH 43606, United States; Promedica Physicians General Surgery, Adrian Charles and Virginia Hickman Hospital, 5640 North Adrian Highway, Adrian, MI 49221, United States

**Keywords:** ileocolic intussusception, Burkitt lymphoma, ileal tumor

## Abstract

Adult intussusception is rare, representing 1% of all small bowel obstructions. The most common cause of adult intussusception is a pathological lead point such as a malignancy. Presenting symptoms can vary, and computed tomography (CT) imaging can aid in the preoperative diagnosis. A 27-year-old male presented to the emergency department with several weeks of diffuse abdominal pain, nausea, and vomiting. A CT scan revealed an abnormal ileocolic configuration with intussusception of the distal ileal loop through the cecum causing a high-grade small bowel obstruction. The patient underwent exploratory laparotomy to relieve the small bowel obstruction caused by the intussusception. A mass was identified intraoperatively which was originating from the terminal ileum. Pathological analysis determined the mass to be Burkitt lymphoma. When adult intussusception is identified, there must be a low index of suspicion for a malignant cause. Burkitt lymphoma was definitively diagnosed after primary resection with histopathology and immunohistochemistry.

## Introduction

Intussusception is the telescoping of a proximal segment of the bowel into an immediately adjacent distal segment. Adult intussusception occurs rarely, representing 1% of all bowel obstructions, 5% of all intussusceptions, and 0.003%–0.02% of all hospital admissions [1]. In contrast to pediatric etiologies, adult intussusception can be associated with a pathological lead point in 80%–90% of symptomatic cases [2]. The following case report describes a patient presenting with ileocolic intussusception that, after surgical resection and pathologic evaluation, was identified as Burkitt lymphoma.

## Case report

A 27-year-old male presented to the emergency department (ED) with complaints of generalized abdominal pain, nausea, and vomiting for 1 month. Within the past 2 weeks, he also reported bright red blood and clots in his stool. His past medical history was unremarkable. His family history was significant for colon cancer in his mother during her twenties. His surgical history included an appendectomy 20 years ago, colonoscopy 3 years ago, and robotic-assisted umbilical hernia repair with mesh 5 months ago. In the ED, his vitals included a heart rate of 67 bpm, blood pressure of 145/85 mmHg, respirations of 16 per minute, SpO2 of 99% on room air, and a temperature of 97.3 F. Physical exam revealed diffuse abdominal tenderness with guarding, but negative for skin changes, distension, rigidity, or palpable masses. The ensuing work-up showed an elevation in white blood cells of 13.3, but CBC otherwise was unremarkable. All other labs including lactate, CRP, magnesium, lipase, CMP, and coagulation studies were unremarkable. The ED provider made the decision to obtain computed tomography (CT) imaging with contrast of the abdomen and pelvis for further evaluation of the abdominal pain.

**Figure 1:**
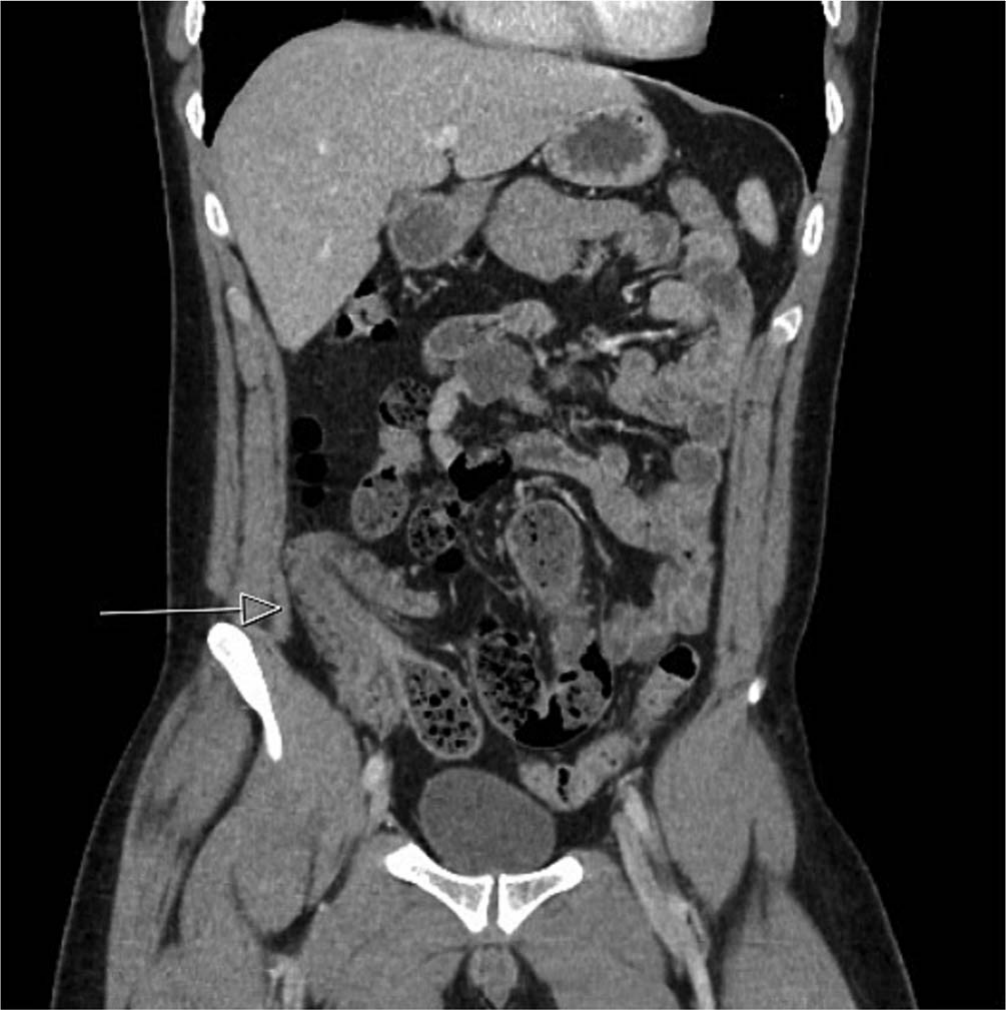
Initial CT imaging, axial cut, showed an abnormal ileocolic configuration with intussusception of the distal ileal loop through the cecum.

**Figure 2:**
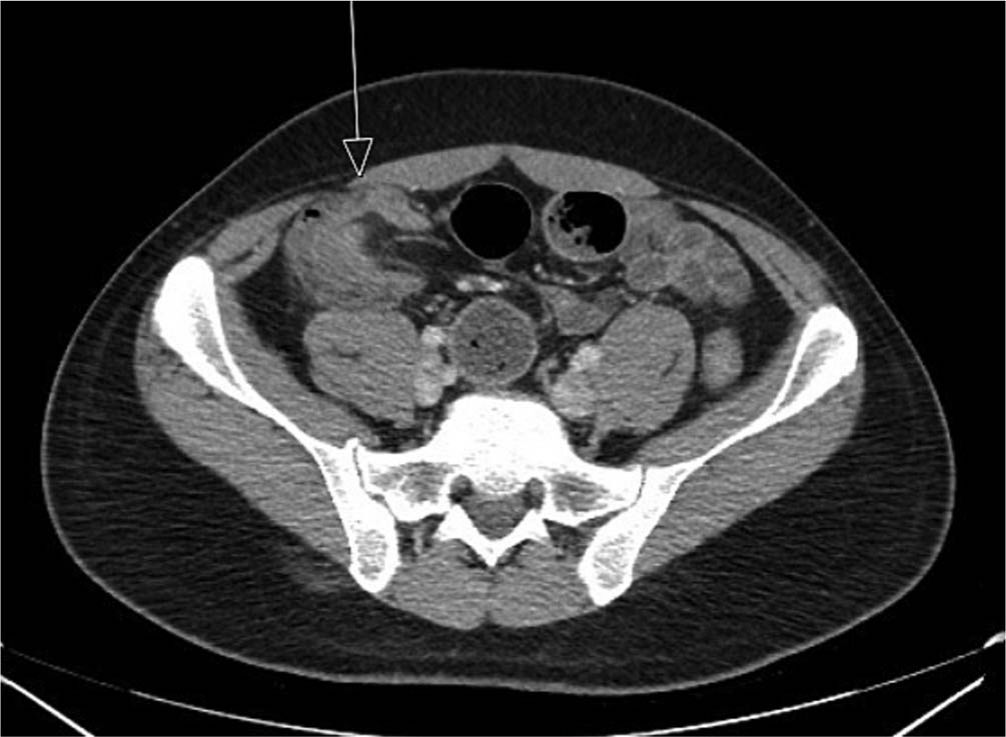
Initial CT imaging, coronal cut, showed an abnormal ileocolic configuration with intussusception of the distal ileal loop through the cecum.

CT imaging (Figs 1 and 2) revealed an abnormal ileocolic configuration with intussusception of the distal ileal loop through the cecum. At this point, the general surgery team was consulted for immediate evaluation. Due to the history, physical exam, and findings identified on CT imaging, the patient was recommended to undergo exploratory laparotomy with intent, at minimum, to reduce the intussusception and likely perform a segmental small bowel resection to address his small bowel obstruction. Right hemicolectomy, however, was also discussed due to the patient’s family history of colon cancer to relieve the intussusception, which was causing a high-grade small bowel obstruction. During surgical exploration, the ileum was fixed within the colon, and the intussusception could not be reduced with gentle manipulation. This area of the ileum was significantly firmer than other areas, which was concerning for an ileal mass. The decision was made to resect this portion of the ileum and perform a right hemicolectomy with ileocolonic side-to-side anastomosis. The specimen was sent to pathology for further investigation. Postoperatively, the patient was hospitalized for 3 days for pain control and monitoring. He recovered in the hospital without further incident and was discharged on day 3 prior to the finalization of his pathology report.

**Figure 3:**
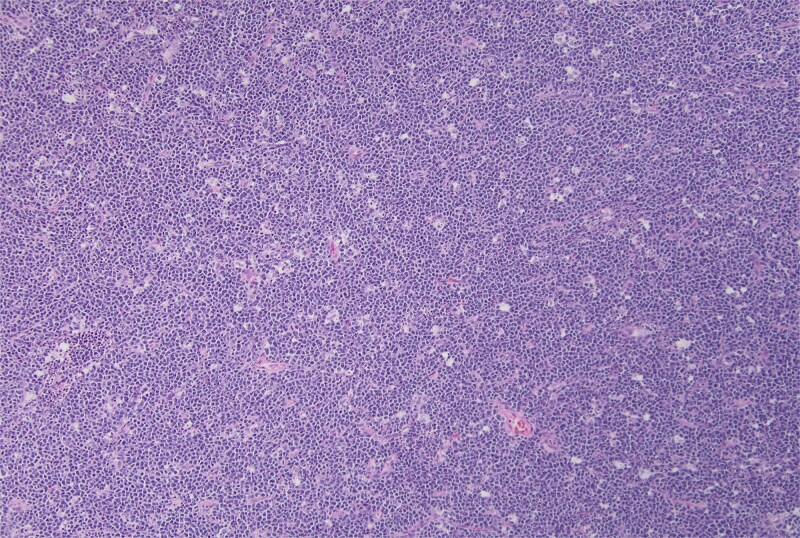
Hematoxylin and eosin stain, starry sky pattern showing a dense infiltrate by Burkitt lymphoma with numerous tingible body macrophages. 10× magnification.

**Figure 4:**
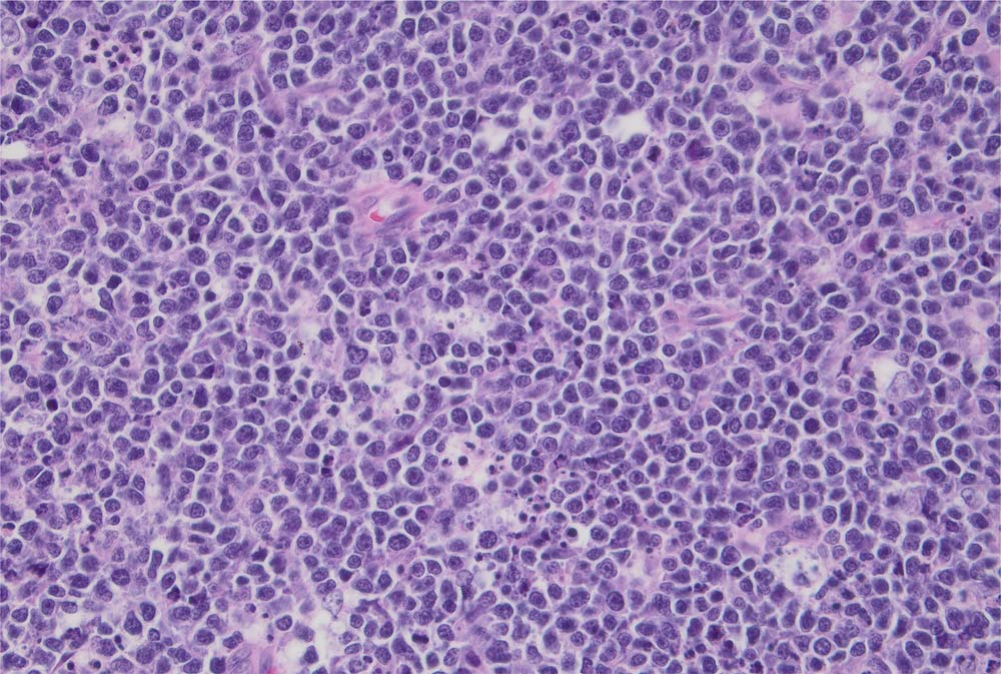
Hematoxylin and eosin stain, high-power view showing a monotonous population of uniform medium-sized lymphocytes and background tingible body macrophages. 40× magnification.

**Figure 5:**
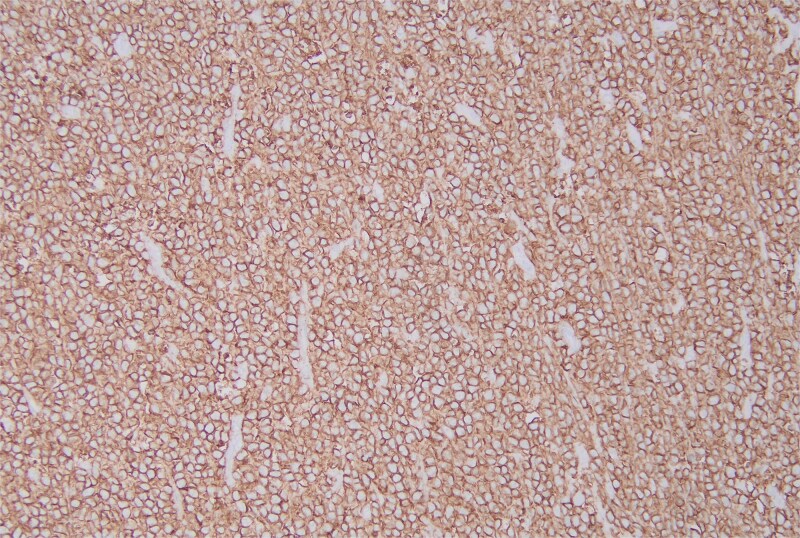
The immunohistochemical stain for CD20 is positive.

**Figure 6:**
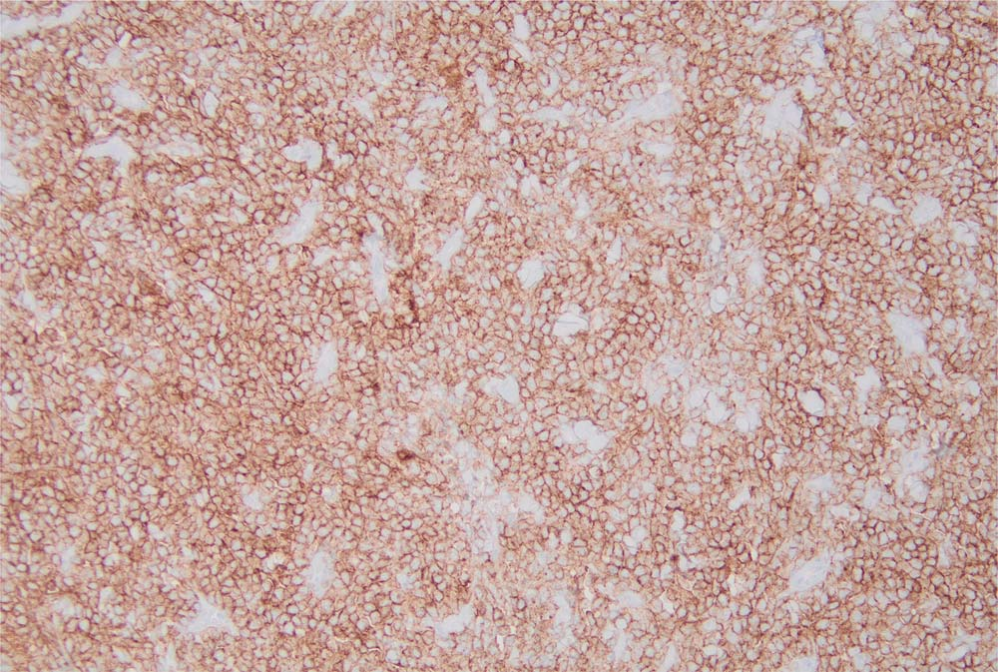
The immunohistochemical stain for CD10 is positive.

**Figure 7:**
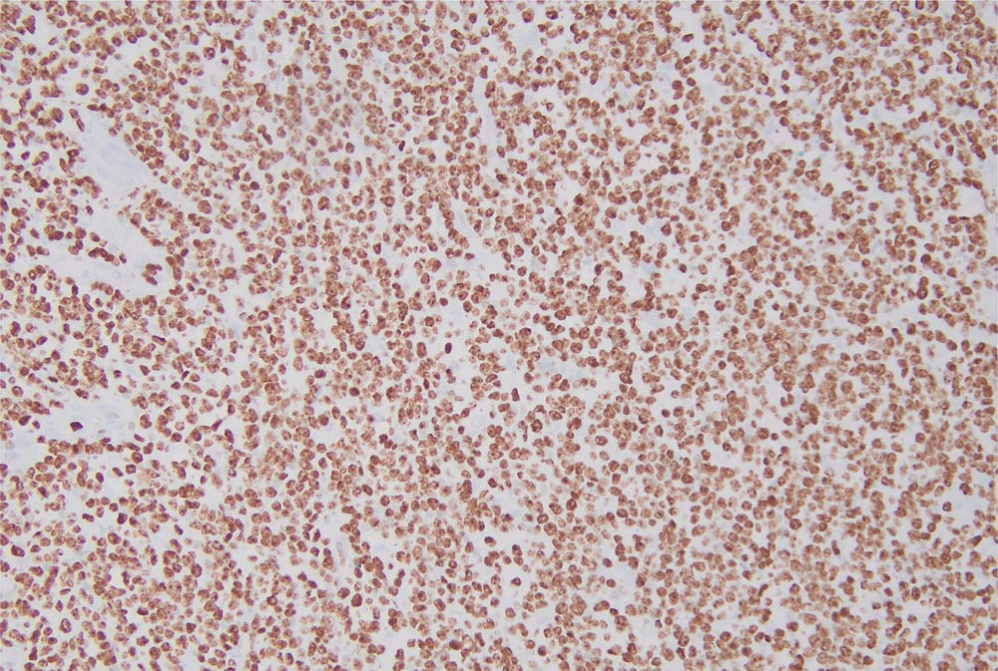
The immunohistochemical stain for Ki-67 is positive.

Pathology report identified a 3.5 × 3.5 × 1.5 cm fungating mass within the ileum along with 33 lymph nodes ranging from 0.2 to 1.5 cm in greatest dimension. Histopathology demonstrated a “starry sky” pattern showing a dense infiltrate by Burkitt lymphoma with numerous tingible body macrophages (Fig. 3) with high-power microscopy confirming a monotonous population of uniform medium-sized lymphocytes and background tingible body macrophages (Fig. 4). All lymph nodes and margins were negative, with the smallest margin measuring 6 cm. Immunostaining showed diffuse positivity for CD20 (Fig. 5), CD10 (Fig. 6), and Ki-67 (Fig. 7). The patient was referred to Oncology for further evaluation.

## Discussion

We report a case of ileocolic intussusception secondary to Burkitt lymphoma in a previously healthy 27-year-old male patient. Intussusception in adult patients is rare and is associated with different etiologies than the more common pediatric presentation [3]. This condition accounts for roughly 1% of intestinal obstructions and in adults is more commonly associated with a pathological lead point in 80%–90% of patients [2, 4].

Ileocolic intussusception is especially noted to be associated with malignancy [2]. Adenocarcinoma tends to be the most common primary malignancy inciting intussusception, and lymphomas have also been reported in addition to metastatic cancers [3]. The nonendemic form of Burkitt lymphoma is a fast-growing and aggressive malignancy that may present as an abdominal mass and is a rare form of intra-abdominal tumor causing intussusception in adults [5].

Presenting symptoms can vary, and adult intussusception often presents with abdominal pain and nausea; presentation may range from acute to chronic [1, 3]. Although early studies had shown that only a small portion of patients were given the diagnosis of intussusception pre-operatively [3], increased utilization of preoperative CT scans seems to have improved diagnostic accuracy; however, an important caveat is the difficulty in determining the etiology of the primary lesion from imaging studies [3, 6].

Surgical management of intussusception in adults is typically centered around resection due to the frequency of malignant pathology in the lead point [2]. In many cases, preoperative reduction of the intussusception in adults is not performed, especially when the lead point is suspected to be malignant [2]. Evaluation of nearby lymph nodes may assist in predicting the probability of malignancy and therefore in the decision to operate without attempting a reduction [7]. Resection is typically a successful intervention, with a 5-year mortality rate of around 90% for localized Burkitt lymphoma [7]. Furthermore, the extent of tumor resection correlates with survival, which is significant given the notable response of Burkitt lymphoma to chemotherapy [5, 8].

## Conclusion

This case presents a 27-year-old male who presented with ileocolic intussusception secondary to an ileal mass that was postoperatively diagnosed as Burkitt lymphoma. Adult intussusception is rare and often secondary to a malignancy. As with many causes of small bowel obstructions, the presenting symptoms are often nonspecific, including abdominal pain, nausea, and vomiting. In patients with preoperative identification of intussusception by CT imaging, it is important to have a high index of suspicion for malignancy. Burkitt lymphoma can only be definitively diagnosed after primary resection and histopathology with immunohistochemistry.
